# Alveolar Abscesses

**Published:** 1922-05

**Authors:** Charles Kingsley Field


### Alveolar Abscesses

BY CHARLES KINGSLEY FIELD, D. M. D.

Demonstrator in Operative Dentistry at Harvard 1913-1914; Orthodontia Clinic at Forsythe Infirmary 1917; American Red Cross Dental Surgeon, overseas, 1918-1919; Professor of Operative Dentistry and Oral Hygiene 1920-1921, Ohio College of Dental Surgery, Cincinnati, Ohio.

Abscesses usually follow the death of the pulp after an incubation period of about four weeks, more or less, by infection of the tissue at the apex of the root.

The germ which is responsible for the infection is Bacillus Pulpae Gangrenosae.

Symptoms in their sequence:—

1. Sensitive to heat,
2. Slight elongation of tooth,
3. Severe throbbing,
4. Extremely sensitive to pressure,
5. Swelling of the gum tissue after the alveolar wall is penetrated,
6. Pain subsides,
7. Swelling spreads,
8. Abscess comes to head,
9. Sometimes breaks through the gum, but lancing is usually necessary.

The pointing of an abscess is usually in the gum buccally nearest to the apex of the tooth affected, this however is not always the case, as abscesses in the upper jaw sometimes break through into the nose, antrum of highmore, floor of the orbit, and abscesses in the lower jaw sometimes break outside the oral cavity, as, on the chin, neck, and sometimes on the chest.

Local results of alveolar abscesses:—

1. Periosteitis or the inflammation of periosteum,
2. Osteitis or the inflammation of bone,
3. Osteo-myelitis or the inflammation of bone marrow,
4. Necrosis or the death of bone, Necrosis is non molecular in its action.

### GINGIVITIS (ULCERATIVE)

A rapidly growing superficial type of infection producing ulceration of the free margin of the gum tissue, which becomes eroded and retracted.

The interproximate tissue is most severely attacked, both labial, buccal and lingual surfaces being affected, and if not checked in time by Prophylactic measures, may seriously affect the alveolar bone.

Vincent's Angina is a highly suppurative infection of this type.

During the Late War a type of gingivitis, known as Trench Mouth, was quite prevalent amongst the armies in Europe:— This particular type was highly putrescent and apparently, also contagious.

STOMATITIS

Inflammation of the mucous membrane of the mouth.

In infants, before the eruption of the temporary teeth and is usually caused by the non-sterilization of the nipples of feeding bottles, by non-pasteurization of the milk and by the mother (when nursing) not having the breasts thoroughly clean.

Later in life Stomatitis may be caused by a great many things.

PYORRHOEA ALVEOLARIS

The above name should be applied only to those forms of pyogenic peridental infection, by which the tissue about the teeth is broken down with the resultant formation of pus. Unfortunately, however, the term has been made current use of in describing almost any form of Gingivitis.

The first man to describe Pyorrhoea was Dr. J. W. Riggs of Hartford, Connecticut. He was of the opinion that it was a purely local infection. It is well to mention here that this conclusion has never been proved either for or against. All of the authorities on this subject have their own opinions and these differ widely.

Dr. G. V. Black's theory agrees with that of Dr. Riggs in that it is purely local. He describes the initial lesion as, an invasion of the peridental membrane by certain infectious organisms which penetrate deeply into the tissues along the so called pericemental glands of Black. The infection thus having gained access to the deeper tissue, sets up inflammation and degenerative changes which spread to the adjacent alveolar process and cause destruction.

There are therefore two distinct stages to Pyorrhoea.

1. The process of infection and degenerative changes.
2. The breaking down of the alveolar bone with the formation of pockets in the interdental spaces with the discharge of pus.

Dr. W. D. Miller has isolated from pyorrhoea pockets, the Staphylococcus Aureus and Albus, the Streptococcus Pyogenes, and has described about sixteen other types of organisms which he was unable to isolate.

Millers conclusions are, that Pyorrhoea may be produced by a wide group of bacteria in varying combinations, and is not caused by any specific type.

The exact cause of Pyorrhoea is still undecided, probably because there is no one cause, however, it is very noticeable that

Pyorrhoea appears always when there is a condition in the mouth which departs considerably from the normal as:—Artificial restorations, Irregular teeth.

Some sources of Pyorrhea follow:—

1. Malocclusion,
2. Gold crowns, when the gold is carried too far under the gum.
3. Fillings which have not been polished on the proximal under the gum.
4. Fillings and crowns which occlude too soon, causing constant traumatic irritation to the root.
5. Seruminal calculus.

In other words, anything which will cause gingivitis is liable to result in Pyorrhoea, provided the particular and varied groups of germs are present to produce the necessary infections.

### SYSTEMIC CAUSES OF PYORRHOEA

*Gout and Arthritis*—In these cases the individual suffers from excessive retention of Uric Acid in the blood and tissues, in direct proportion to the severity of the diseases.

The resistance of the peridental tissue is proportionately lowered, thereby making it more subject to infection and predisposed to peridental disease.

Diabetic patients are especially subject to Pyorrhoea.

Pyorrhoea very seldom appears in the young and vigorous, because their power of resistance is extremely high.

### DR. HOWE'S THEORY OF DIET IN RELATION TO PYORRHOEA

Dr. Percy Howe of the Research Department of Forsythe Infirmary, Boston, gave a lecture in 1920 before the Massachusetts Dental Society, on the subject of Diet in relation to Pyorrhoea.

After experimenting on guinea pigs by innoculating them with pus from the Pyorrhoea pocket of a human subject; he was unable to produce Pyorrhoea;—but by changing the diet by not feeding them any green food (rich in vitamines) he was able to produce extremely advanced stages of Pyorrhoea, having all the symptoms of the human disease, such as pus pockets, loosening of the teeth, and receeding gums.

Dr. Howe therefore concludes that, Diet, especially as it relates to Vitamines, is of very great importance in the consideration of the causes of Pyorrhoea.

According to Dr. Howe, a meat diet which has little or no Vitamines will cause Pyorrhoea, and a vegetable diet which is rich in Vitamines will prevent Pyorrhoea.

There is room for considerably more investigation along this line.

